# Evolution of H7N9 highly pathogenic avian influenza virus in the context of vaccination

**DOI:** 10.1080/22221751.2024.2343912

**Published:** 2024-04-17

**Authors:** Yujie Hou, Guohua Deng, Pengfei Cui, Xianying Zeng, Bin Li, Dongxue Wang, Xinwen He, Cheng Yan, Yaping Zhang, Jiongjie Li, Jinming Ma, Yanbing Li, Xiurong Wang, Guobin Tian, Huihui Kong, Lijie Tang, Yasuo Suzuki, Jianzhong Shi, Hualan Chen

**Affiliations:** a State Key Laboratory for Animal Disease Control and Prevention, Harbin Veterinary Research Institute, CAAS, Harbin, People’s Republic of China; b College of Veterinary Medicine, Northeast Agricultural University, Harbin, People’s Republic of China; c Institute of Western Agriculture, CAAS, Changji, People's Republic of China; d Department of Medical Biochemistry, University of Shizuoka School of Pharmaceutical Sciences, Shizuoka, Japan; e National Poultry Laboratory Animal Resource Center, Harbin Veterinary Research Institute, CAAS, Harbin, People’s Republic of China

**Keywords:** Avian influenza virus; H7N9; evolution; receptor-binding properties; pathogenicity; antigenicity

## Abstract

Human infections with the H7N9 influenza virus have been eliminated in China through vaccination of poultry; however, the H7N9 virus has not yet been eradicated from poultry. Carefully analysis of H7N9 viruses in poultry that have sub-optimal immunity may provide a unique opportunity to witness the evolution of highly pathogenic avian influenza virus in the context of vaccination. Between January 2020 and June 2023, we isolated 16 H7N9 viruses from samples we collected during surveillance and samples that were sent to us for disease diagnosis. Genetic analysis indicated that these viruses belonged to a single genotype previously detected in poultry. Antigenic analysis indicated that 12 of the 16 viruses were antigenically close to the H7-Re4 vaccine virus that has been used since January 2022, and the other four viruses showed reduced reactivity with the vaccine. Animal studies indicated that all 16 viruses were nonlethal in mice, and four of six viruses showed reduced virulence in chickens upon intranasally inoculation. Importantly, the H7N9 viruses detected in this study exclusively bound to the avian-type receptors, having lost the capacity to bind to human-type receptors. Our study shows that vaccination slows the evolution of H7N9 virus by preventing its reassortment with other viruses and eliminates a harmful characteristic of H7N9 virus, namely its ability to bind to human-type receptors.

## Introduction

Influenza A viruses pose threats to animal and human health [1–7]. They are classified into different subtypes based on the antigenicity of their haemagglutinin (HA) and neuraminidase (NA) proteins. Currently, the H1-H16 and N1-N9 subtypes have been detected in avian species [8], whereas the H17N10 and H18N11 subtypes have been identified in bats [9,10]. Viruses of the H1N1, H2N2, and H3N2 subtypes have caused influenza pandemics [11–13], and avian influenza viruses of several other subtypes have also spilled over from avian species and caused human infections [14–21]. While viruses of other subtypes have only occasionally caused human infections, viruses of the H5 and H7 subtypes have respectively caused 971 and 1687 human infections globally [22,23], causing a threat to public health that cannot be ignored.

Certain H5 and H7 avian influenza viruses are highly pathogenic to poultry and can cause up to 100% mortality in susceptible birds that are not vaccinated. The H5 viruses have led to the loss of 506 million poultry around the world since 2005 [24], and over 311 million poultry were lost between January 2020 and December 2023 in response to outbreaks mainly caused by H5 avian influenza viruses bearing the clade 2.3.4.4b HA gene. It is worth noting that most poultry losses occurred in countries in Europe and North America, as well as Japan and South Korea, where “stamping-out” strategies have been implemented for highly pathogenic avian influenza control, and that the damage to the poultry industry has been relatively light in countries where vaccination strategies have been adopted. Vaccination is now recommended by the World Organization for Animal Health and being considered by a growing number of countries, including the United States of America [25–27].

The H7N9 viruses that emerged in China in early 2013 were low pathogenic in avian species and mainly circulated in chickens in live poultry markets in southern China [28,29]. Despite tremendous efforts to control the virus, including disinfecting poultry markets and culling birds in H7N9 virus-positive poultry markets, the H7N9 virus continued to circulate and caused over 1560 human infections over five waves from February 2013 to September 2017. Moreover, the virus acquired basic amino acids in its HA cleavage site that allowed it to become highly pathogenic in chickens in early 2017, causing several influenza outbreaks in poultry in China [29]. To control H7N9 viruses, a bivalent H5/H7 inactivated vaccine was developed and has used in poultry in China since September 2017 [30]. The HA gene of the H7N9 vaccine seed virus has been updated three times to ensure the antigenicity of the vaccine matches the target virus [31–34]. Application of the vaccine in poultry has successfully prevented human infections with H7N9 virus, although the virus has not been eliminated from poultry [30,35].

Eradicating highly pathogenic avian influenza virus through vaccination alone will take longer than the “stamping-out” strategy because virus in a contaminated environment may infect and replicate silently in immunocompromised poultry, raising concerns that viruses that spread silently in vaccinated poultry would evolve faster and be more dangerous to humans [36,37]. H5 highly pathogenic viruses are widely circulating in wild birds and poultry in many countries that have different control strategies, and it is therefore difficult to use H5 virus to show how vaccination affects virus evolution. However, H7N9 virus is mainly circulating in China, and analysis of the virus detected after the implementation of the vaccination strategy allows us to see the evolution of the virus and improve the understanding of the effectiveness of vaccination strategies for highly pathogenic avian influenza control.

In this study, we detected 16 H7N9 viruses from samples we collected during our routine surveillance and samples that were submitted to our laboratory for diagnosis between January 2020 and June 2023. We extensively analyzed these viruses to investigate their genetic evolution, receptor-binding properties, virulence in mice and chickens, and antigenic properties. Our findings provide important insights into the evolution of highly pathogenic influenza viruses in the context of vaccination, and clarify some common misconceptions about vaccination strategies for highly pathogenic avian influenza control in poultry.

## Results

### H7N9 viruses detected in poultry between January 2020 and June 2023

During our routine surveillance, we collected swab samples from 160,359 poultry in live poultry markets, slaughterhouses, and poultry farms between January 2020 and June 2023, and isolated five H7N9 viruses: two from samples collected in a slaughterhouse in Liaoning province in May 2020, two from samples collected in a live poultry market in Shandong province in April 2021, and one from a sample collected in a live poultry market in Yunnan province in April 2021 (Table 1).

**Table 1.** H7N9 viruses detected in this study.

Sample information				Virus	
Date	Type	Source/Province		Name (Abbreviation)	HA cleavage site
May 8, 2020	Swab	Slaughterhouse/Liaoning		A/chicken/Liaoning/S1506/2020 (CK/LN/S1506/20)	-KRKRTAR/G-
May 8, 2020	Swab	Slaughterhouse/Liaoning		A/chicken/Liaoning/S1534/2020 (CK/LN/S1534/20)	-KRKRTAR/G-
November 10, 2020	Swab	Farm/Hebei		A/chicken/Hebei/SD004/2020 (CK/HeB/SD004/20)	-KRKRTAR/G-
March 21, 2021	Lung	Farm/Shandong		A/chicken/Shandong/SD020/2021 (CK/SD/SD020/21)	-KRKRTAR/G-
April 1, 2021	Swab	Poultry market/Yunnan		A/chicken/Yunnan/S1400/2021 (CK/YN/S1400/21)	-KRKRIAR/G-
April 17, 2021	Lung	Farm/Yunnan		A/chicken/Yunnan/SD024/2021 (CK/YN/SD024/21)	-KRKRIAR/G-
April 21, 2021	Swab	Poultry market/Shandong		A/chicken/Shandong/S1346/2021 (CK/SD/S1346/21)	-KRKRTAR/G-
April 21, 2021	Swab	Poultry market/Shandong		A/chicken/Shandong/S1360/2021 (CK/SD/S1360/21)	-KRKRTAR/G-
March 25, 2022	Lung	Farm/Hebei		A/chicken/Hebei/SD003/2022 (CK/HeB/SD003/22)	-KKKRTAR/G-
May 4, 2022	Lung	Farm/Shandong		A/chicken/Shandong/SD010/2022 (CK/SD/SD010/22)	-KRKRTAR/G-
May 25, 2022	Swab	Farm/Hebei		A/chicken/Hebei/SD011/2022 (CK/HeB/SD011/22)	-KRKRTAR/G-
June 4, 2022	Swab	Farm/Shandong		A/chicken/Shandong/SD013/2022 (CK/SD/SD013/22)	-KRKRTAR/G-
September 21, 2022	Lung	Farm/Hebei		A/chicken/Hebei/SD022/2022 (CK/HeB/SD022/22)	-KRKRTAR/G-
February 7, 2023	Lung	Farm/Hebei		A/chicken/Hebei/SD004/2023 (CK/HeB/SD004/23)	-KRKRAAR/G-
February 10, 2023	Lung	Farm/Hebei		A/chicken/Hebei/SD016/2023 (CK/HeB/SD016/23)	-KKKRTAR/G-
April 14, 2023	Lung	Farm/Hebei		A/chicken/Hebei/SD020/2023 (CK/HeB/SD020/23)	-KKKRIAR/G-

We also isolated 11 H7N9 viruses from swab or lung samples from sick or dead chickens from different poultry farms that were submitted to our laboratory: one from Yunnan province in 2021, three from Shandong provinces (one in 2021 and two in 2022), and seven from Hebei province (one in 2020, three in 2022, and three in 2023) (Table 1).

### Phylogenic analysis of highly pathogenic H7N9 viruses

We previously reported that highly pathogenic H7N9 and H7N2 viruses detected in China from February 2017 to January 2018 formed nine genotypes (G1-G9) [30], and the viruses detected between February 2018 and December 2019 formed two genotypes (G2 and G10) [32]. To understand the evolution and genetic relationship of the H7N9 viruses detected in this study, we sequenced the genomes of the 16 H7N9 viruses [the sequences have been deposited in the Global Initiative on Sharing All Influenza Data database (GISAID; https://www.gisaid.org); accession numbers are EPI3220049-3220176], and compared them with those of representative viruses from the genotypes we reported previously [29,30,32].

**Figure 1.**
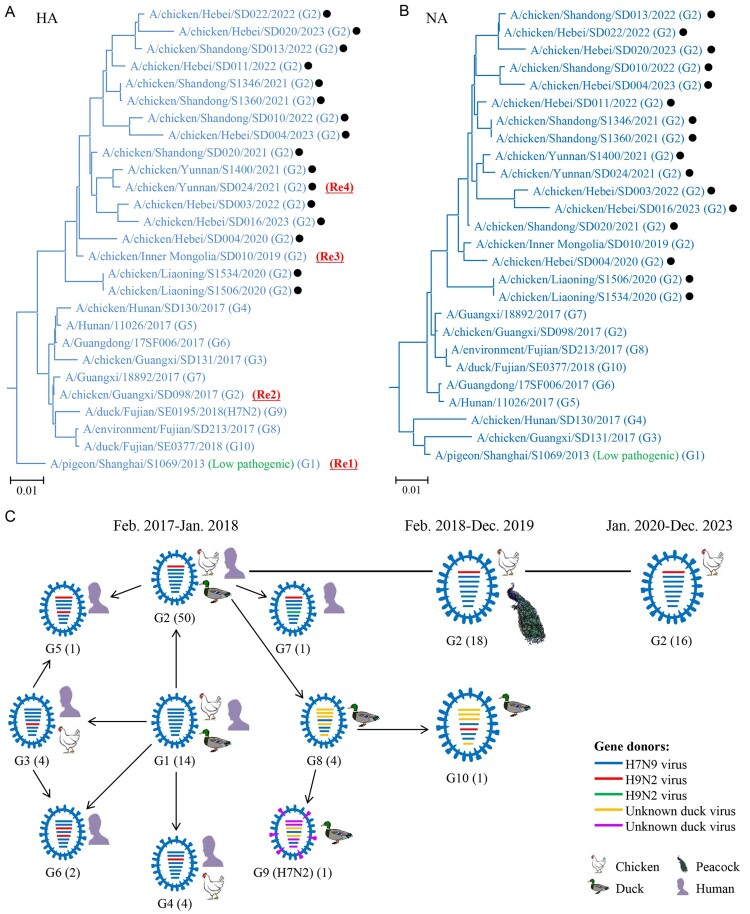
Phylogenetic analyses and genotypes of H7N9 viruses. The phylogenetic trees of the HA (a) and NA (b) genes were rooted to A/chicken/Rostock/45/1934 (H7N1) and A/chicken/Italy/22A/1998 (H5N9), respectively. The H7N9 strains reported in this study are marked with black dots. The four H7N9 viruses that provided surface genes for the H7-Re1, H7-Re2, H7-Re3, and H7-Re4 vaccine seed strains are shown in red in parentheses. (c) Genotypes of H7N9 viruses and hosts in which these genotypes were detected. The numbers of strains for each genotype in each time period are provided in parentheses. The genotypes of the viruses isolated between February 2017 and December 2019 were reported previously [30,32].

The HA gene of the 16 H7N9 viruses shared identity of 95.2%–100% at the nucleotide level, and we detected five different amino acid motifs at their HA cleavage sites: –PKRKRTAR/G-, –PKRKRIAR/G-, –PKRKRAAR/G-, –PKKKRTAR/G-, and – PKKKRIAR/G– (Table 1). The NA, basic polymerase 2 (PB2), basic polymerase 1 (PB1), acidic polymerase (PA), nucleoprotein (NP), matrix (M), and nonstructural protein (NS) genes of the 16 H7N9 viruses shared 95.1%–100%, 96.1%–99.9%, 96.5%–100%, 96.6%–100%, 96.9%–100%, 96.4%–100%, and 97.0%–99.9% identity, respectively, at the nucleotide level. In the phylogenetic trees of these eight genes, the viruses detected in this study closely clustered (Figure 1(a,b), and Figure S1), and all eight genes of the 16 viruses shared greater than 95% identity with the previously reported genotype 2 (G2) virus (Figure 1(c)) [29,30,32]. These results suggest that H7N9 viruses detected in chickens in recent years have not reassorted with other avian influenza viruses to form new genotypes.

### Replication and virulence of the H7N9 viruses in mice

**Figure 2.**
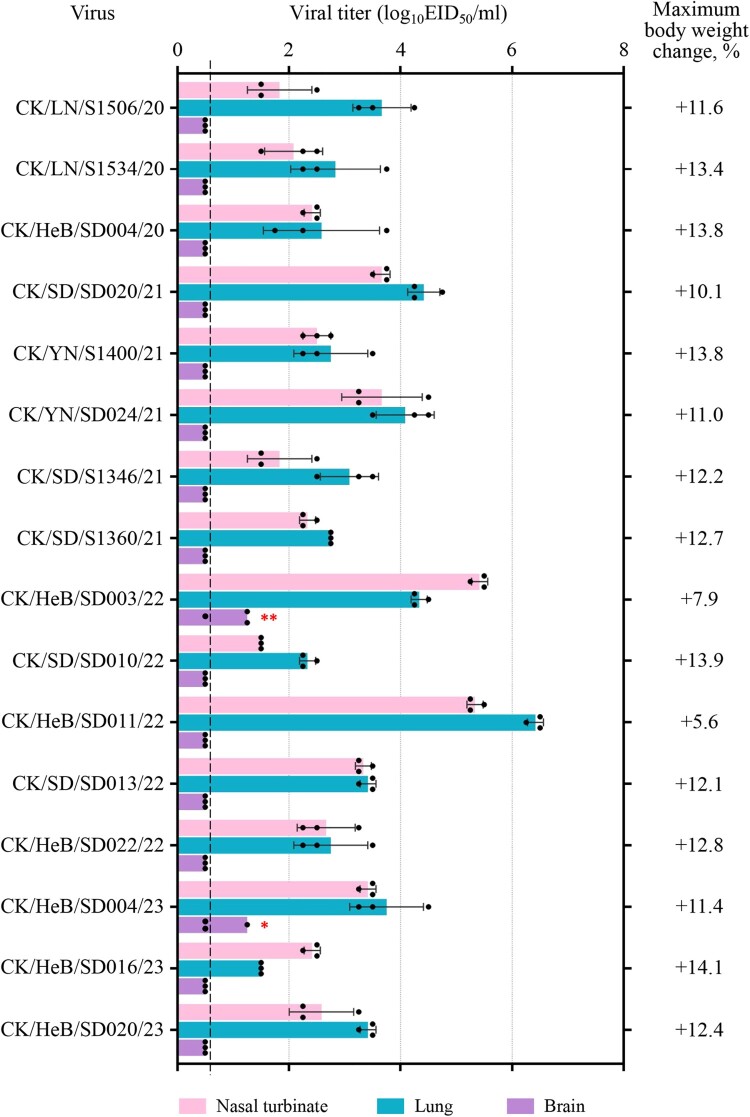
Replication and virulence of H7N9 viruses in mice. Virus titres in organs of mice inoculated i.n. with 10^6^ EID_50_ of different H7N9 viruses. Three mice from each group were euthanized and their organs were collected on Day 3 p.i. for virus titration in eggs. Data shown are means ± standard deviations. The values labelled with one red star indicate that the virus was detected in the organ of only one mouse, and two red stars indicate that the virus was detected in the organ of two mice. The dashed lines indicate the lower limit of detection. The maximum body weight change in the groups of five mice after inoculation with 10^6^ EID_50_ of the test virus are shown on the right side.

Previous studies showed that the H7N9 highly pathogenic viruses isolated between 2017 and 2019 have different pathogenicity in mice, meaning that some strains are highly lethal to mice with a 50% mouse lethal dose (MLD_50_) value of 3.2 log_10_ 50% egg infectious doses (EID_50_), and some strains cannot kill any mice even at a dose of 10^6^ EID_50_ [30,32]. To investigate the pathogenicity of the H7N9 viruses detected in this study, we inoculated groups of eight 6-week-old female BALB/c mice intranasally (i.n.) with 10^6^ EID_50_ of test viruses. Three mice in each group were euthanized on Day 3 post-inoculation (p.i.) and their organs were harvested for virus titration in eggs. The remaining five mice in each group were observed for two weeks for body weight change and death. We found that all 16 viruses replicated in the nasal turbinates and lungs of mice, with viral titres ranging from 1.5 to 5.4 log_10_EID_50_ in the nasal turbinates and from 1.5 to 6.4 log_10_EID_50_ in the lungs (Figure 2). CK/HeB/SD004/23 and CK/HeB/SD003/22 were respectively detected in the brain of one mouse and two mice; no virus was detected in the spleen or kidneys of any of the inoculated mice (Figure 2). All of the mice survived, and maximum body weight increases ranged from 5.6% to 14.1% at the end of two-week observation period (Figure 2). These results demonstrate that H7N9 viruses detected in recent years are nonlethal to mice.

### Receptor-binding property analysis

**Figure 3.**
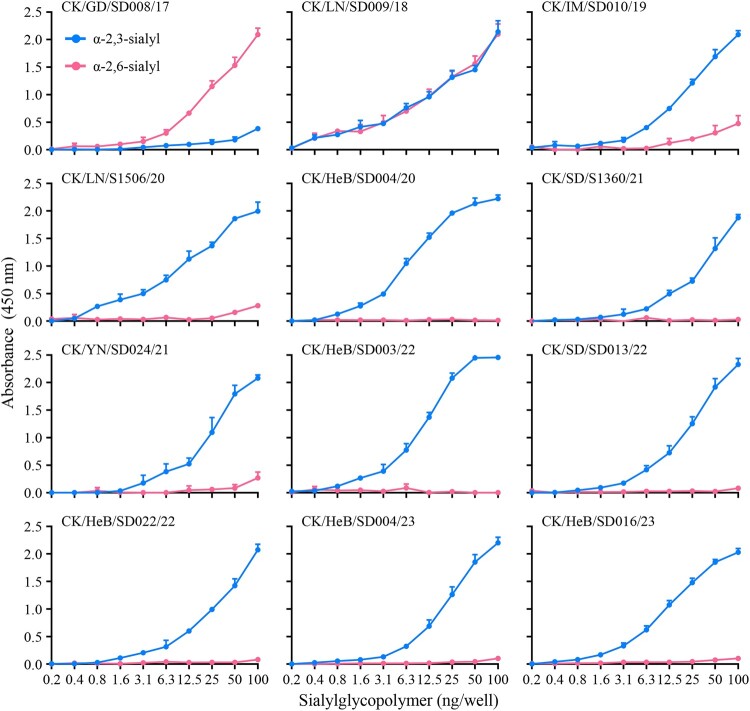
Receptor-binding properties of H7N9 viruses. The receptor-binding properties of H7N9 viruses are detected with sialylglycopolymers (α−2,3-sialylglycopolymer, blue; α−2,6-sialylglycopolymer, red). The data shown are the means of three repeats; the error bars indicate standard deviations.

Binding to human-type receptors is a prerequisite for a novel influenza virus to efficiently transmit from human to human [38–46]. Previous studies indicate that the low pathogenic H7N9 viruses circulating in China from 2013 to 2016 bound to α2,6-linked sialic acids (human-type receptor) with high affinity and to α2,3-linked sialic acids (avian-type receptor) with low affinity [5,47,48], and that the receptor-binding properties of the highly pathogenic H7N9 avian influenza viruses isolated between 2017 and 2019 varied among strains [29,32]. To investigate the receptor-binding properties of the H7N9 viruses in this study, we selected nine viruses that were isolated at different times and evaluated their affinity for two different glycopolymers (α−2, 3 and α−2, 6-sialylglycopolymer) that represent the avian-type and the human-type receptor, respectively. We also included three early viruses, CK/GD/SD008/17, CK/LN/SD009/18, and CK/IM/SD010/19, for comparison. Similar to previous reports, CK/GD/SD008/17 bound to the human-type receptor with high affinity and to the avian-type receptor with low affinity, CK/LN/SD009/18 bound to the avian-type receptor and the human-type receptor with similar affinity, and CK/IM/SD010/19 bound to the avian-type receptor with high affinity and to the human-type receptor with low affinity (Figure 3) [32]. However, the nine H7N9 viruses detected in this study bound to the avian-type receptor with high affinity, but did not bind to the human-type receptor (Figure 3). These results indicate that H7N9 highly pathogenic viruses have gradually lost their ability to bind to the human-type receptor.

### Replication and virulence of H7N9 viruses in chickens

The H7N9 highly pathogenic avian influenza viruses we detected previously have an intravenous pathogenicity index (IVPI) value of 3.0 [29] and kill chickens infected intranasally at a dose of 10^6^EID_50_ [29,32]. The five H7N9 viruses we isolated during the surveillance were all from apparently healthy chickens, and although the other 11 viruses were isolated occasionally from sick or dead birds on different farms, large-scale disease or death of chickens was not reported. Moreover, different motifs were observed at the HA cleavage site of these viruses (Table 1) and it is unclear whether these newly detected viruses have altered pathogenicity in chickens. We therefore selected and tested six representative viruses (Table 2), which were isolated at different times and had different motifs at the HA cleavage site, in chickens. All six viruses were highly pathogenic in chickens based on their IVPI values [a virus with an IVPI value ≥1.2 is considered highly pathogenic [49] ], since they all killed chickens within 48 h of inoculation, yielding IVPI values of 2.9–3.0 (0 = least pathogenic; 3 = most pathogenic) (Table 2).

**Table 2.** Replication and virulence of H7N9 viruses in chickens.

Virus	Motif at the HA cleavage site	Intravenous pathogenicity index (IVPI)	Virus shedding on Day 3 post inoculation (*p*.i.): mean titre, log_10_ EID_50_/ml (positive/total)^a^		Death/ Total
			Pharynx	Cloacae	
CK/HeB/SD004/20	-KRKRTAR/G-	3.0	4.3 (11/11)	4.6 (11/11)	4/8
CK/YN/SD024/21	-KRKRIAR/G-	2.9	4.1 (11/11)	4.0 (11/11)	8/8
CK/HeB/SD022/22	-KRKRTAR/G-	3.0	3.5 (11/11)	4.3 (11/11)	5/8
CK/HeB/SD004/23	-KRKRAAR/G-	3.0	4.8 (11/11)	3.6 (11/11)	8/8
CK/HeB/SD016/23	-KKKRTAR/G-	2.9	3.6 (11/11)	3.6 (11/11)	5/8
CK/HeB/SD020/23	-KKKRIAR/G-	3.0	4.3 (11/11)	3.4 (11/11)	7/8

Notes: a: Groups of 11 six-week-old SPF chickens were inoculated i.n. with 0.1 ml of 10^6^ EID_50_ of test viruses. Pharyngeal and cloacal swabs of all birds were collected on day 3 *p*.i., three chickens in each group were then euthanized for testing the viral titres in organs as shown in Figure 4, the remaining eight chickens were observed for death for two weeks.

**Figure 4.**
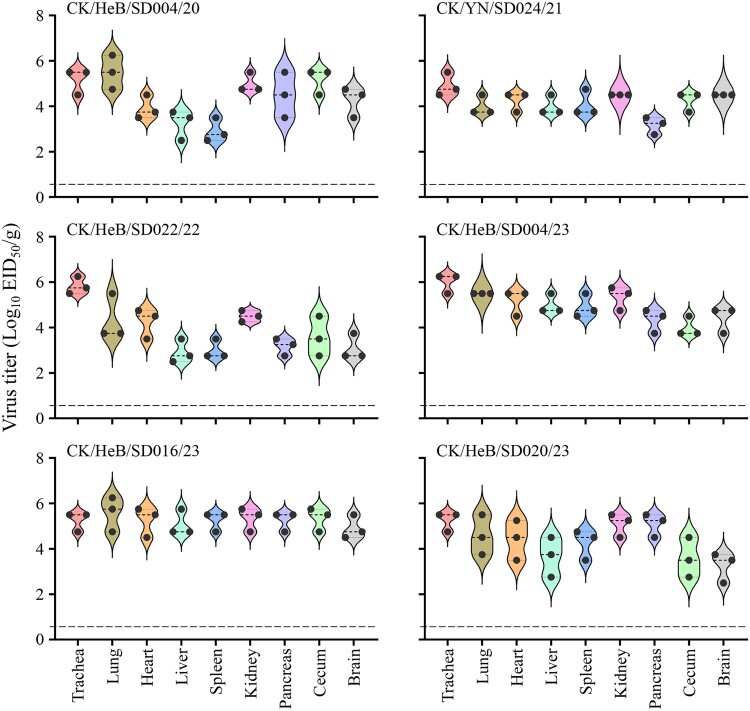
Viral titres in organs of chickens intranasally infected with H7N9 virus. Groups of three 6-week-old SPF chickens were intranasally inoculated with 0.1 ml of inoculum containing 10^6^ EID_50_ of the indicated virus. The chickens were euthanized on Day 3 post-inoculation and their organs were collected for viral titration in eggs.

We also inoculated 11 SPF chickens intranasally (i.n.) with 10^6^EID_50_ of test virus to investigate virus shedding, replication in organs, and lethality. We found that chickens inoculated with any of the six strains shed virus through both the pharynx and cloacae (Table 2), and virus could be detected in all of the organs tested of the three chickens in each group that were euthanized on Day 3 post-inoculation (p.i.) (Figure 4). Chickens inoculated with the CK/YN/SD024/21 or CK/HeB/SD004/23 all died during the two-week observation period, but four, five, five, and seven of the eight chickens that were infected with CK/HeB/SD004/20, CK/HeB/SD022/22, CK/HeB/SD016/23, and CK/HeB/SD020/23, respectively, died (Table 2). These results indicate that, although the H7N9 viruses detected in this study had IVPI values greater than 2.9, some of them showed reduced pathogenicity in chickens upon intranasal infection.

### Antigenic analysis

**Figure 5.**
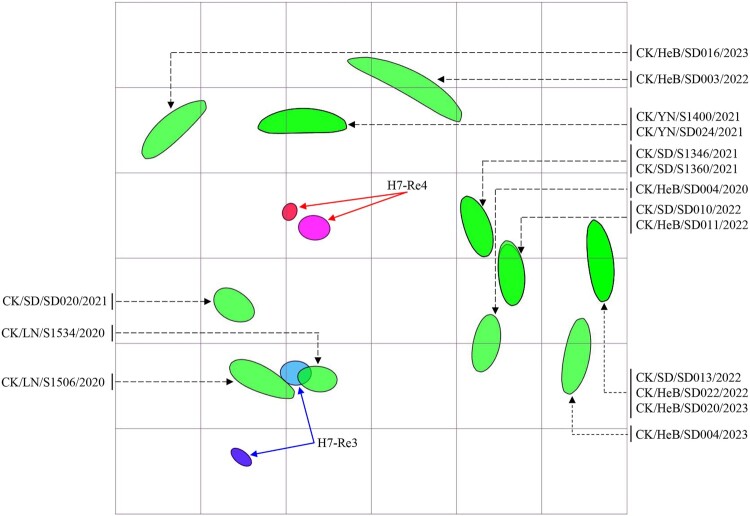
Antigenic cartography of H7N9 viruses. The antigenic map was generated using the HI assay data shown in Table S1. Each unit in the coordinates represents a 2-fold difference in the HI titre. The ovals coloured red and dark blue represent the antisera generated from the H7-Re3 and H7-Re4 vaccine strains, respectively. The ovals coloured pink and light blue show the viruses H7-Re3 and H7-Re4, respectively. The ovals coloured green represent the test viruses.

An H7-Re3 vaccine strain bearing the HA gene of the H7N9 virus CK/IM/SD010/2019(H7N9) was used for H7N9 control in poultry between July 2020 and December 2021 [22]. The vaccine seed virus was replaced by H7-Re4 bearing the HA gene of CK/YN/SD024/2021(H7N9) in January 2022 [33]. To understand the antigenic relevance of the H7-Re3 and H7-Re4 vaccines to the 16 viruses detected in this study, we evaluated the cross-reactivity of H7-Re3 and H7-Re4 antisera against these viruses in a haemagglutinin inhibition (HI) assay. The H7-Re3 and H7-Re4 antisera antibody titres against homologous viruses were 512 and 1024, respectively (Figure 5, Table S1). Four viruses reacted well with H7-Re3 antiserum, with HI titres 2– to 8-fold lower than that to the homologous titre; the other 12 viruses reacted with the H7-Re3 antiserum relatively poor, with titres 16- to 32-fold lower than that of the homologous titre (Figure 5, Table S1). Twelve viruses reacted well with H7-Re4 antiserum with HI titres 2- to 8-fold lower than that to the homologous titre, and four viruses reacted with the H7-Re4 antiserum with titres 16-fold lower than that of the homologous titre (Figure 5, Table S1). These results indicate that most of the H7N9 viruses detected in this study cross-react well with antiserum induced by the H7-Re4 vaccine, but a few viruses had reduced reactivity with the H7-Re4 antiserum.

### Key amino acid changes that contribute to the antigenic difference between H7-Re3 and H7-Re4

**Figure 6.**
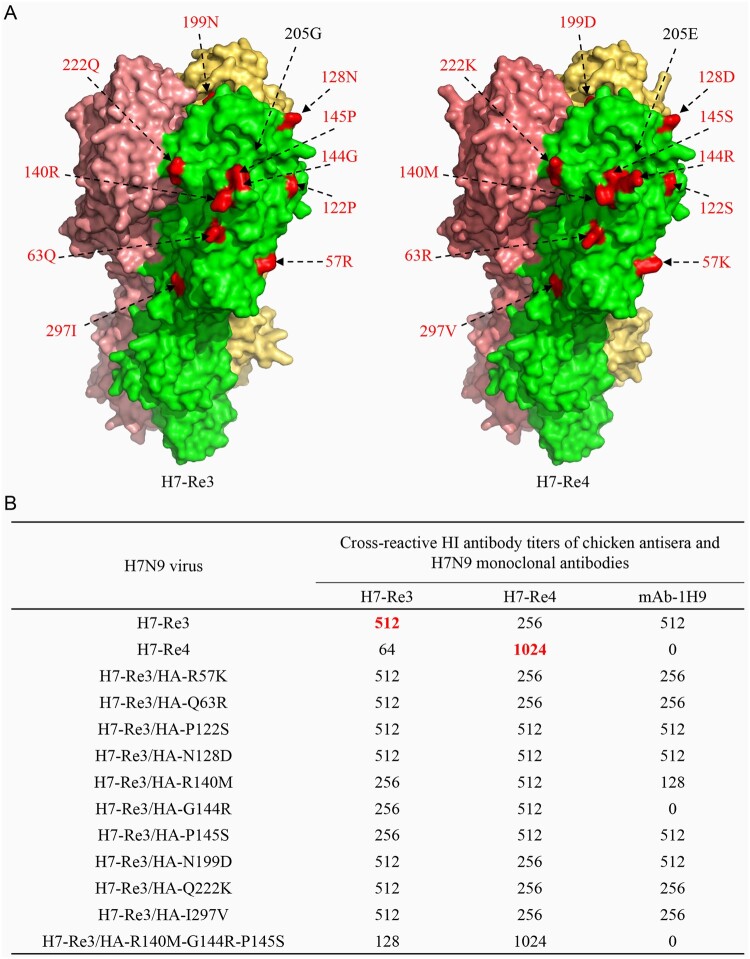
Key mutations in HA1 that contribute to the antigenic difference between the H7-Re3 and H4-Re4 viruses. (a) The amino acid differences in the HA1 between the H7-Re3 and H7-Re4 vaccine strains are shown in the 3D structure of the HA1 protein. The amino acid residues located on the surface of the globular head of the HA1 protein are shown in red. (b) Cross-reactive HI antibody titres of different H7-Re3 mutants against H7-Re3 antiserum, H7-Re4 antiserum, and an H7N9 monoclonal antibody.

As shown in Table S1, the cross-reactive HI antibody titre of H7-Re4 against the antiserum induced by H7-Re3 was 8-fold lower than that to the homologous virus, and the cross-reactive HI antibody titre of H7-Re3 against the antiserum induced by H7-Re4 virus was 4-fold lower than that to the homologous virus (Table S1). There are 11 amino acid differences in the HA1 of these two viruses, and 10 of these 11 amino acids (i.e. all but the one at position 205) are located on the surface of the globular head of the HA1 protein (Figure 6(a)). We therefore investigated which amino acid(s) change may have contributed to the observed antigenic difference between these two viruses.

Using H7-Re3 as a backbone, we generated 10 mutants by reverse genetics, each of which contained an amino acid substitution that appeared in H7-Re4 HA (Figure 6(b)). We then evaluated their reactivity to antisera induced by H7-Re3 and H7-Re4, respectively. We also tested the reactivity of the mutants to the monoclonal antibody mAb-1H9, which reacts with the H7-Re3 virus but not with the H7-Re4 virus (Figure 6(b)). We found that seven mutants reacted like H7-Re3 to the H7-Re3 antiserum, whereas the HI titres of three mutants –H7-Re3/HA-R140M, H7-Re3/HA-G144R, and H7-Re3/HA-P145S– were 2-fold lower than that of the homologous virus. Five mutants reacted similarly to H7-Re3 to the H7-Re4 antiserum, whereas the HI titres of the other five mutants to the H7-Re4 antiserum were 2-fold higher than that of the H7-Re3 virus (Figure 6(b)). Mutant H7-Re3/HA-G144R did not react with the monoclonal antibody mAb-1H9, mutant H7-Re3/HA-R140M reacted with mAb-1H9 with a titre 4-fold lower than that of the H7-Re3 virus, four mutants reacted with mAb-1H9 with titres 2-fold lower than that of the H7-Re3 virus, and four mutants reacted with mAb-1H9 with titres similar to that of the H7-Re3 virus (Figure 6(b)). Since three mutants carrying amino acid substitutions at position 140, 144, or 145 all had reduced reactivity with H7-Re3 antiserum but increased reactivity with the H7-Re4 antiserum compared with that of the H7-Re3 virus, we generated a mutant (H7-Re3/HA-R140M-G144R-P145S) that contained the above three amino acid changes in HA1 and found that its reactivity to the H7-Re3 antiserum was 4-fold lower than that of the homologous H7-Re3 virus, and its cross-reactive titre with the H7-Re4 antiserum was the same as that of the H7-Re4 virus, which was 4-fold higher than that of the H7-Re3 virus (Figure 6(b)). Of note, H7-Re3/HA-R140M-G144R-P145S did not react with the mAb-1H9 (Figure 6(b)). These results suggest that the antigenic difference between H7-Re3 and H7-Re4 is mainly due to amino acid substitutions at positions 140, 144, and 145 in HA.

## Discussion

Vaccination has been a strategy for H7N9 avian influenza control in China since September 2017, and human infections with H7N9 virus have been completely prevented through this strategy, even though previous studies [30,32] and the current study indicate that the H7N9 virus has not been eliminated from poultry in China. Analysis of the H7N9 viruses detected from chickens between January 2020 and June 2023 revealed that only one genotype of H7N9 viruses still exist in chickens in four provinces in China. We found that the viruses are nonlethal in mice, and some of the strains have reduced lethality in chickens when administered intranasally. Importantly, the H7N9 viruses have lost their affinity for the human-type receptor, a characteristic that is required for human-to-human transmission of avian influenza virus [42,43].

The IVPI value is used to classify the pathogenicity of avian influenza virus, and when the IVPI value is equal to or greater than 1.2, the virus is considered to be highly pathogenic [49]. Based on this criterion, all of the H7N9 viruses with the insertion of extra amino acids at the HA cleavage site that were reported previously [32,50,51] and those detected in this study are highly pathogenic strains; however, with intranasal inoculation of a dose of 10^6^EID_50_, four of the six viruses we tested in this study did not kill all eight chickens, suggesting that some H7N9 viruses have reduced pathogenicity to chickens. Of note, the reduced virulence of these viruses appeared to be independent of their motif in the cleavage site of HA. Previous studies have indicated that changes of key amino acids in the NS1 gene reduce the pathogenicity of H5N1 viruses in chickens [52,53]; the genetic change responsible for the pathogenicity change of the H7N9 viruses remains to be determined.

When two or more different influenza viruses infect the same host, the viruses may exchange gene segments and form viruses of new genotypes, and such reassortment is an important mechanism of influenza virus evolution. The currently circulating H5N1 viruses bearing the clade 2.3.4.4b HA gene reassorted with other influenza viruses and formed numerous genotypes in Europe and North America in less than two years [54–59], and the H7N9 highly pathogenic avian influenza viruses reassorted with other viruses and formed seven different genotypes of H7N9 viruses and an H7N2 virus in less than a year after their emergence at the beginning of 2017 [30]. A previous study [32] and this study indicate that only one genotype (G2) of the H7N9 virus has been detected in poultry in China since March 2018, suggesting that even if vaccination does not completely eradicate the H7N9 highly pathogenic avian influenza virus from poultry in a short period of time, vaccination still prevented its reassortment with other viruses, thereby significantly slowing its evolution.

The early H7N9 viruses bound to human-type receptors with high affinity and to avian-type receptors with low affinity [5,47,48], which is a major reason why these viruses easily infected humans. However, since 2018, the receptor-binding preference of the H7N9 viruses we detected from poultry gradually changed: their affinity for avian-type receptors has gradually increased, and their affinity for human-type receptors has gradually declined [32]. The viruses detected in this study exclusively bound to avian-type receptors. Although the underlying mechanism that has driven this receptor-binding change remains to be investigated, the fact that the surviving H7N9 viruses in vaccinated poultry have lost a key harmful trait indicates that vaccination does not cause the highly pathogenic avian influenza virus to become more dangerous to humans.

In conclusion, our study reveals the genetic evolution and biological properties of the H7N9 highly pathogenic avian influenza virus detected in recent years in China. Although antigenic differences could be easily detected between the surviving viruses and the vaccine seed strain, the lack of reassortment with other viruses and the loss of human-type receptor binding ability of existing H7N9 viruses strongly suggest that although vaccination alone cannot eradicate highly pathogenic H7N9 influenza viruses from poultry in a short time, surviving H7N9 viruses are not evolving faster or becoming more dangerous to humans.

## Materials and methods

### Ethics statements and facility

Swab samples collected during active surveillance were processed at the enhanced biosafety level 2 (BSL2+) facility of the Harbin Veterinary Research Institute of the Chinese Academy of Agricultural Sciences (HVRI, CAAS). All experiments with live H7N9 viruses or lungs harvested from dead birds were carried out at the animal biosafety level 3 (ABSL3) facility in HVRI, CAAS. The study was carried out strictly in accordance with the recommendations of the Guide for the Care and Use of Laboratory Animals of the Ministry of Science and Technology of China. The protocol for the animal studies was approved by the Committee on the Ethics of Animal Experiments of the HVRI, CAAS.

### Sample collection and virus isolation

Swab samples were collected from birds in live poultry markets, poultry farms, and slaughterhouses during active surveillance, and the oropharyngeal and cloacal swab of the same bird was placed in a tube and counted as one sample. Lung samples from dead birds were homogenized as described previously [60]. All individual samples were inoculated into 10-day-old embryonated chicken eggs and incubated for 48 h at 37°C. The HA subtype was identified by using the HI test, and the NA subtype was confirmed by sequence analysis. The H7N9 viruses were biologically cloned three times by limiting dilution in specific-pathogen-free (SPF) embryonated eggs. Virus stocks were cultured in SPF chicken eggs and kept at −70°C.

### Genetic and phylogenetic analyses

The genomes of the H7N9 viruses detected in this study were sequenced on an Applied Biosystems DNA analyzer (3500xL Genetic Analyzer, United States of America). Their nucleotide sequences were edited using the Seqman module of the DNAStar package. Phylogenetic analysis of the surface genes and the six internal genes of the H7N9 viruses was performed using the MEGA 11.0.13 software package, implementing the neighbor-joining method, and evaluated by 1000 bootstrap analyses. The groups of each gene segment in the phylogenetic trees were classified using 95% sequence identity cutoffs.

### Receptor-binding properties of H7N9 viruses

The receptor-binding specificity of the H7N9 viruses was determined using a solid phase direct binding test, with two glycopolymers: α−2, 3-sialylglycopolymer [Neu5Acα2-3Galb1-4GlcNAcb1-pAP (para-aminophenyl)-alpha-polyglutamic acid (α-PGA)] (avian-type receptor) and α−2, 6-sialylglycopolymer [Neu5Acα2-6Galb1-4GlcNAcb1-pAP (para-aminophenyl)-alpha-polyglutamic acid (α-PGA)] (human-type receptor), which were synthesized by Yamasa Corporation Co. Ltd, Japan. In this study, chicken antiserum against the H7N9 virus (CK/YN/SD024/21) was generated and used as the primary antibody. Horseradish peroxidase (HRP) conjugated goat-anti-chicken antibody (Sigma-Aldrich, St. Louis, MO, USA) was used as the secondary antibody. The absorbance was determined at a wavelength of 450 nm.

### Replication and virulence of H7N9 viruses in mice

Groups of eight 6-week-old female BALB/c mice (Beijing Experimental Animal Center, Beijing, China) were lightly anaesthetized with CO_2_ and then 10^6^ EID_50_ of the H7N9 virus in a volume of 50 μl was inoculated i.n. Three mice in each group were euthanized on Day 3 p.i. and their organs, including brain, nasal turbinates, spleen, kidneys, and lungs, were collected for virus titration in chicken eggs. The virus titres were calculated using the method of Reed and Muench. The remaining five mice were monitored daily for weight change and mortality for up to two weeks.

### Replication and virulence of H7N9 viruses in chickens

To determine the IVPI value of the H7N9 viruses, groups of ten 6-week-old SPF White Leghorn chickens housed in isolator cages were inoculated intravenously with 0.1 ml of a 1:10 dilution of bacterium-free allantoic fluid containing virus and were observed for signs of disease or death for 10 days.

To investigate the replication and virulence of the viruses, groups of 11 six-week-old SPF White Leghorn chickens were inoculated i.n. with 0.1 ml of 10^6^ EID_50_ of test viruses. Pharyngeal and cloacal swabs of all birds were collected on Day 3 p.i., and then three birds in each group were killed and their organs (trachea, lungs, heart, liver, spleen, kidneys, pancreas, caecum, and brain) were collected for virus titration in chicken eggs. The virus titres were calculated using the method of Reed and Muench. The remaining eight chickens were observed for mortality for two weeks.

### Antigenic analyses

Antigenic analysis of the H7N9 viruses was carried out using the HI test. To generate the antisera, groups of 6-week-old SPF chickens were inoculated with 0.5 ml of oil-emulsified inactivated viruses, and their sera were harvested three weeks later. The two H7N9 vaccine seed viruses bear the internal genes of the A/Puerto Rico/8/1934 (H1N1) (PR8) virus, but their HA and NA genes were derived from different H7N9 viruses: H7-Re3 contains surface genes derived from CK/IM/SD010/19, whereas H7-Re4 contains surface genes derived from CK/YN/SD024/21. The generation of these vaccine seed viruses was reported previously [22,33]. Antigenic map of the viruses was obtained using Antigenic Cartography software (http://www.antigenic-cartography.org/).

### Three-dimensional structural simulation and analysis of HA proteins

The 3D structure of the HA protein was simulated using SWISS-MODEL software. The image of the HA protein was drawn using Pymol software as previously described [32]. Amino acid differences between the two vaccine strains are shown on the surface of the globular head of the HA1 protein.

### Virus rescue

H7N9-Re3 mutants were generated by reverse genetics. Mutations were introduced into the HA gene by PCR using the QuickChange Site-Directed Mutagenesis kit; the primer sequences are available upon request. The rescued viruses were fully sequenced to ensure the absence of unwanted mutations.

## Supplementary Material

updated_Supporting_figure_S1

Hou_Table_S1

## References

[1] Meng F, Chen Y, Song Z, et al. Continued evolution of the Eurasian avian-like H1N1 swine influenza viruses in China. Sci China Life Sci. 2023 Feb;66(2):269–282. doi:10.1007/s11427-022-2208-0 36219302

[2] Cui P, Shi J, Yan C, et al. Analysis of avian influenza A (H3N8) viruses in poultry and their zoonotic potential, People’s Republic of China, September 2021 to May 2022. Euro Surveill. 2023 Oct;28(41):pii = 2200871. 10.2807/1560-7917.ES.2023.28.41.2200871 PMC10571489 37824247

[3] Xing X, Shi J, Cui P, et al. Evolution and biological characterization of H5N1 influenza viruses bearing the clade 2.3.2.1 hemagglutinin gene. Emerg Microbes Infect. 2024;13(1):2284294. doi:10.1080/22221751.2023.2284294 37966008 PMC10769554

[4] Liu L, Yang H, Guo F, et al. Emergence of H5N1 highly pathogenic avian influenza in Democratic People's Republic of Korea. J Integr Agric. 2022;21(5):1534–1538. doi:10.1016/S2095-3119(21)63829-7

[5] Zhang Q, Shi J, Deng G, et al. H7N9 influenza viruses are transmissible in ferrets by respiratory droplet. Science. 2013;341(6144):410–414. doi:10.1126/science.1240532 23868922

[6] Zhao Y, Zhao Y, Liu S, et al. Phylogenetic and epidemiological characteristics of H9N2 avian influenza viruses in Shandong Province, China from 2019 to 2021. J Integr Agric. 2023;22(3):881–896. doi:10.1016/j.jia.2022.08.114

[7] Liu K, Qi X, Bao C, et al. Novel H10N3 avian influenza viruses: a potential threat to public health. Lancet Microbe. 2024 Jan 31: S2666–S5247. (23)00409-3. doi:10.1016/S2666-5247(23)00409-3 38309285

[8] Scheibner D, Ulrich R, Fatola OI, et al. Variable impact of the hemagglutinin polybasic cleavage site on virulence and pathogenesis of avian influenza H7N7 virus in chickens, turkeys and ducks. Sci Rep. 2019;9(1):11556. doi:10.1038/s41598-019-47938-3 31399610 PMC6689016

[9] Tong S, Li Y, Rivailler P, et al. A distinct lineage of influenza A virus from bats. Proc Natl Acad Sci U S A. 2012;109(11):4269–4274. doi:10.1073/pnas.1116200109 22371588 PMC3306675

[10] Tong S, Zhu X, Li Y, et al. New world bats harbor diverse influenza A viruses. PLoS Pathog. 2013;9(10):e1003657. doi:10.1371/journal.ppat.1003657 24130481 PMC3794996

[11] Taubenberger JK, Reid AH, Krafft AE, et al. Initial genetic characterization of the 1918 “Spanish” influenza virus. Science. 1997;275(5307):1793–1796. doi:10.1126/science.275.5307.1793 9065404

[12] de Jong JC, Claas EC, Osterhaus AD, et al. A pandemic warning? Nature. 1997;389(6651):554. doi:10.1038/39218 9335492 PMC7095477

[13] Laver WG, Air GM, Dopheide TA, et al. Amino acid sequence changes in the haemagglutinin of A/Hong Kong (H3N2) influenza virus during the period 1968–77. Nature. 1980;283(5746):454-7. doi:10.1038/283454a0 6153236

[14] Bao P, Liu Y, Zhang X, et al. Human infection with a reassortment avian influenza A H3N8 virus: an epidemiological investigation study. Nat Commun. 2022;13(1):6817. doi:10.1038/s41467-022-34601-1 36357398 PMC9649012

[15] Subbarao K, Klimov A, Katz J, et al. Characterization of an avian influenza A (H5N1) virus isolated from a child with a fatal respiratory illness. Science. 1998;279(5349):393–396. doi:10.1126/science.279.5349.393 9430591

[16] Gu W, Shi J, Cui P, et al. Novel H5N6 reassortants bearing the clade 2.3.4.4b HA gene of H5N8 virus have been detected in poultry and caused multiple human infections in China. Emerg Microbes Infect. 2022;11(1):1174–1185. doi:10.1080/22221751.2022.2063076 35380505 PMC9126593

[17] Tweed SA, Skowronski DM, David ST, et al. Human illness from avian influenza H7N3, British Columbia. Emerg Infect Dis. 2004;10(12):2196–2199. doi:10.3201/eid1012.040961 15663860 PMC3323407

[18] Fouchier RA, Schneeberger PM, Rozendaal FW, et al. Avian influenza A virus (H7N7) associated with human conjunctivitis and a fatal case of acute respiratory distress syndrome. Proc Natl Acad Sci U S A. 2004;101(5):1356–1361. doi:10.1073/pnas.0308352100 14745020 PMC337057

[19] Gao R, Cao B, Hu Y, et al. Human infection with a novel avian-origin influenza A (H7N9) virus. N Engl J Med. 2013;368(20):1888–1897. doi:10.1056/NEJMoa1304459 23577628

[20] Peiris M, Yuen KY, Leung CW, et al. Human infection with influenza H9N2. Lancet. 1999;354(9182):916–917. doi:10.1016/S0140-6736(99)03311-5 10489954

[21] Qi X, Qiu H, Hao S, et al. Human infection with an avian-origin influenza A (H10N3) virus. N Engl J Med. 2022;386(11):1087–1088. doi:10.1056/NEJMc2112416 35294820

[22] Shi J, Zeng X, Cui P, et al. Alarming situation of emerging H5 and H7 avian influenza and effective control strategies. Emerg Microbes Infect. 2023;12(1):2155072. doi:10.1080/22221751.2022.2155072 36458831 PMC9754034

[23] WHO. Influenza at the human-animal interface summary and assessment. Geneva: World Health Organization. 2023. https://www.who.int/teams/global-influenza-programme/avian-influenza.

[24] OIE-WAHIS. Latest animal disease events. Paris: World Animal Health Information System. 2023: https://wahis.woah.org.

[25] Cohen J. Bird shots. Science. 2023 Apr 7;380(6640):24–27. doi:10.1126/science.adi1004 37023176

[26] Stokstad E. Wrestling with bird flu, Europe considers once-taboo vaccines. Science. 2022;376(6594):682–683. doi:10.1126/science.adc9450 35549419

[27] Wille M, Barr IG. Resurgence of avian influenza virus. Science. 2022;376(6592):459–460. doi:10.1126/science.abo1232 35471045

[28] Shi J, Deng G, Liu P, et al. Isolation and characterization of H7N9 viruses from live poultry markets — Implication of the source of current H7N9 infection in humans. Chinese Sci Bull. 2013;58(16):1857–1863. doi:10.1007/s11434-013-5873-4

[29] Shi J, Deng G, Kong H, et al. H7N9 virulent mutants detected in chickens in China pose an increased threat to humans. Cell Res. 2017;27(12):1409–1421. doi:10.1038/cr.2017.129 29151586 PMC5717404

[30] Shi J, Deng G, Ma S, et al. Rapid evolution of H7N9 highly pathogenic viruses that emerged in China in 2017. Cell Host Microbe. 2018;24(4):558–568. doi:10.1016/j.chom.2018.08.006 30269969 PMC6310233

[31] Zeng X, Chen X, Wu J, et al. Protective efficacy of an H5/H7 trivalent inactivated vaccine produced from Re-11, Re-12, and H7-Re2 strains against challenge with different H5 and H7 viruses in chickens. J Integr Agric. 2020;19(9):2294–2300. doi:10.1016/S2095-3119(20)63301-9

[32] Yin X, Deng G, Zeng X, et al. Genetic and biological properties of H7N9 avian influenza viruses detected after application of the H7N9 poultry vaccine in China. PLoS Pathog. 2021;17(4):e1009561. doi:10.1371/journal.ppat.1009561 33905456 PMC8104392

[33] Zeng X, He X, Meng F, et al. Protective efficacy of an H5/H7 trivalent inactivated vaccine (H5-Re13, H5-Re14, and H7-Re4 strains) in chickens, ducks, and geese against newly detected H5N1, H5N6, H5N8, and H7N9 viruses. J Integr Agric. 2022;21(7):2086–2094. doi:10.1016/S2095-3119(22)63904-2

[34] Zhao Y, Chen P, Hu Y, et al. Recombinant duck enteritis virus bearing the hemagglutinin genes of H5 and H7 influenza viruses is an ideal multivalent live vaccine in ducks. Emerg Microbes Infect. 2024;13(1):2284301. doi:10.1080/22221751.2023.2284301 37966272 PMC10769552

[35] Zeng X, Tian G, Shi J, et al. Vaccination of poultry successfully eliminated human infection with H7N9 virus in China. Sci China Life Sci. 2018;61(12):1465–1473. doi:10.1007/s11427-018-9420-1 30414008

[36] Gambotto A, Barratt-Boyes SM, de Jong MD, et al. Human infection with highly pathogenic H5N1 influenza virus. Lancet. 2008;371(9622):1464–1475. doi:10.1016/S0140-6736(08)60627-3 18440429

[37] Iwami S, Suzuki T, Takeuchi Y. Paradox of vaccination: is vaccination really effective against avian flu epidemics? PLoS One. 2009;4(3):e4915. doi:10.1371/journal.pone.0004915 19295921 PMC2657368

[38] Liu Q, Zhou B, Ma W, et al. Analysis of recombinant H7N9 wild-type and mutant viruses in pigs shows that the Q226L mutation in HA is important for transmission. J Virol. 2014;88(14):8153–8165. doi:10.1128/JVI.00894-14 24807722 PMC4097782

[39] Qu Z, Ma S, Kong H, et al. Identification of a key amino acid in hemagglutinin that increases human-type receptor binding and transmission of an H6N2 avian influenza virus. Microbes Infect. 2017;19(12):655–660. doi:10.1016/j.micinf.2017.09.008 28951329

[40] Wang Z, Yang H, Chen Y, et al. A single-amino-acid substitution at position 225 in hemagglutinin alters the transmissibility of Eurasian avian-like H1N1 swine influenza virus in Guinea pigs. J Virol. 2017 Nov 1;91(21):e00800–17. 28814518 10.1128/JVI.00800-17 PMC5640871

[41] Zhang Y, Zhao C, Hou Y, et al. Pandemic threat posed by H3N2 avian influenza virus. Sci China Life Sci. 2021;64(11):1984–1987. doi:10.1007/s11427-021-1916-4 33765225

[42] Herfst S, Schrauwen EJ, Linster M, et al. Airborne transmission of influenza A/H5N1 virus between ferrets. Science. 2012;336(6088):1534–1541. doi:10.1126/science.1213362 22723413 PMC4810786

[43] Imai M, Watanabe T, Kiso M, et al. A highly pathogenic avian H7N9 influenza virus isolated from A human Is lethal in some ferrets infected via respiratory droplets. Cell Host Microbe. 2017;22(5):615–626.e8. doi:10.1016/j.chom.2017.09.008 29056430 PMC5721358

[44] Matrosovich M, Tuzikov A, Bovin N, et al. Early alterations of the receptor-binding properties of H1, H2, and H3 avian influenza virus hemagglutinins after their introduction into mammals. J Virol. 2000;74(18):8502–8512. doi:10.1128/JVI.74.18.8502-8512.2000 10954551 PMC116362

[45] Vines A, Wells K, Matrosovich M, et al. The role of influenza A virus hemagglutinin residues 226 and 228 in receptor specificity and host range restriction. J Virol. 1998;72(9):7626–7631. doi:10.1128/JVI.72.9.7626-7631.1998 9696865 PMC110023

[46] Zhang Y, Zhang Q, Kong H, et al. H5N1 hybrid viruses bearing 2009/H1N1 virus genes transmit in Guinea pigs by respiratory droplet. Science. 2013;340(6139):1459–1463. doi:10.1126/science.1229455 23641061

[47] Xiong X, Martin SR, Haire LF, et al. Receptor binding by an H7N9 influenza virus from humans. Nature. 2013;499(7459):496–499. doi:10.1038/nature12372 23787694

[48] Dortmans JC, Dekkers J, Wickramasinghe IN, et al. Adaptation of novel H7N9 influenza A virus to human receptors. Sci Rep. 2013;3:3058. doi:10.1038/srep03058 24162312 PMC3808826

[49] WOAH. Manual of diagnostic tests and vaccines for terrestrial animals. Vol. 12th ed. Paris: World Organization for Animal Health. 2023. https://www.woah.org/en/home/

[50] Yu D, Xiang G, Zhu W, et al. The re-emergence of highly pathogenic avian influenza H7N9 viruses in humans in mainland China, 2019. Euro Surveill. 2019 May;24(21):1900273. 31138362 10.2807/1560-7917.ES.2019.24.21.1900273 PMC6540644

[51] Zhao B, Wang W, Song Y, et al. Genetic characterization and pathogenicity of H7N9 highly pathogenic avian influenza viruses isolated from South China in 2017. Front Microbiol. 2023;14:1105529. doi:10.3389/fmicb.2023.1105529 36960283 PMC10027924

[52] Li Z, Jiang Y, Jiao P, et al. The NS1 gene contributes to the virulence of H5N1 avian influenza viruses. J Virol. 2006;80(22):11115–11123. doi:10.1128/JVI.00993-06 16971424 PMC1642184

[53] Zhu Q, Yang H, Chen W, et al. A naturally occurring deletion in its NS gene contributes to the attenuation of an H5N1 swine influenza virus in chickens. J Virol. 2008;82(1):220–228. doi:10.1128/JVI.00978-07 17942562 PMC2224367

[54] Cui P, Shi J, Wang C, et al. Global dissemination of H5N1 influenza viruses bearing the clade 2.3.4.4b HA gene and biologic analysis of the ones detected in China. Emerg Microbes Infect. 2022;11(1):1693–1704. doi:10.1080/22221751.2022.2088407 35699072 PMC9246030

[55] Tian J, Bai X, Li M, et al. Highly pathogenic avian influenza virus (H5N1) clade 2.3.4.4b introduced by wild birds, People’s Republic of China, 2021. Emerg infect Dis. 2023 Jul;29(7):1367–1375. doi:10.3201/eid2907.221149 37347504 PMC10310395

[56] Domanska-Blicharz K, Swieton E, Swiatalska A, et al. Outbreak of highly pathogenic avian influenza A(H5N1) clade 2.3.4.4b virus in cats, Poland, June to July 2023. Euro Surveill. 2023 Aug;28(31):2300366. doi:10.2807/1560-7917.ES.2023.28.31.2300366 37535474 PMC10401911

[57] Lindh E, Lounela H, Ikonen N, et al. Highly pathogenic avian influenza A(H5N1) virus infection on multiple fur farms in the South and Central Ostrobothnia regions of Finland, July 2023. Euro Surveill. 2023 Aug;28(31):2300400. doi:10.2807/1560-7917.ES.2023.28.31.2300400 37535475 PMC10401912

[58] Pohlmann A, Stejskal O, King J, et al. Mass mortality among colony-breeding seabirds in the German Wadden Sea in 2022 due to distinct genotypes of HPAIV H5N1 clade 2.3.4.4b. J Gen Virol. 2023 Apr;104(4):001834. doi:10.1099/jgv.0.001834 37014781

[59] Youk S, Torchetti MK, Lantz K, et al. H5N1 highly pathogenic avian influenza clade 2.3.4.4b in wild and domestic birds: introductions into the United States and reassortments, December 2021-April 2022. Virology. 2023 Oct;587:109860. doi:10.1016/j.virol.2023.109860 37572517

[60] Cui P, Zeng X, Li X, et al. Genetic and biological characteristics of the globally circulating H5N8 avian influenza viruses and the protective efficacy offered by the poultry vaccine currently used in China. Sci China Life Sci. 2022 Apr;65(4):795–808. doi:10.1007/s11427-021-2025-y 34757542

