# Supplementary figures and images for: Evolution of H7N9 highly pathogenic avian influenza virus in the context of vaccination
Source: Emerg Microbes Infect. 2024 Apr 17;13(1):2343912. doi: 10.1080/22221751.2024.2343912 (PMC11060016; doi:10.1080/22221751.2024.2343912)

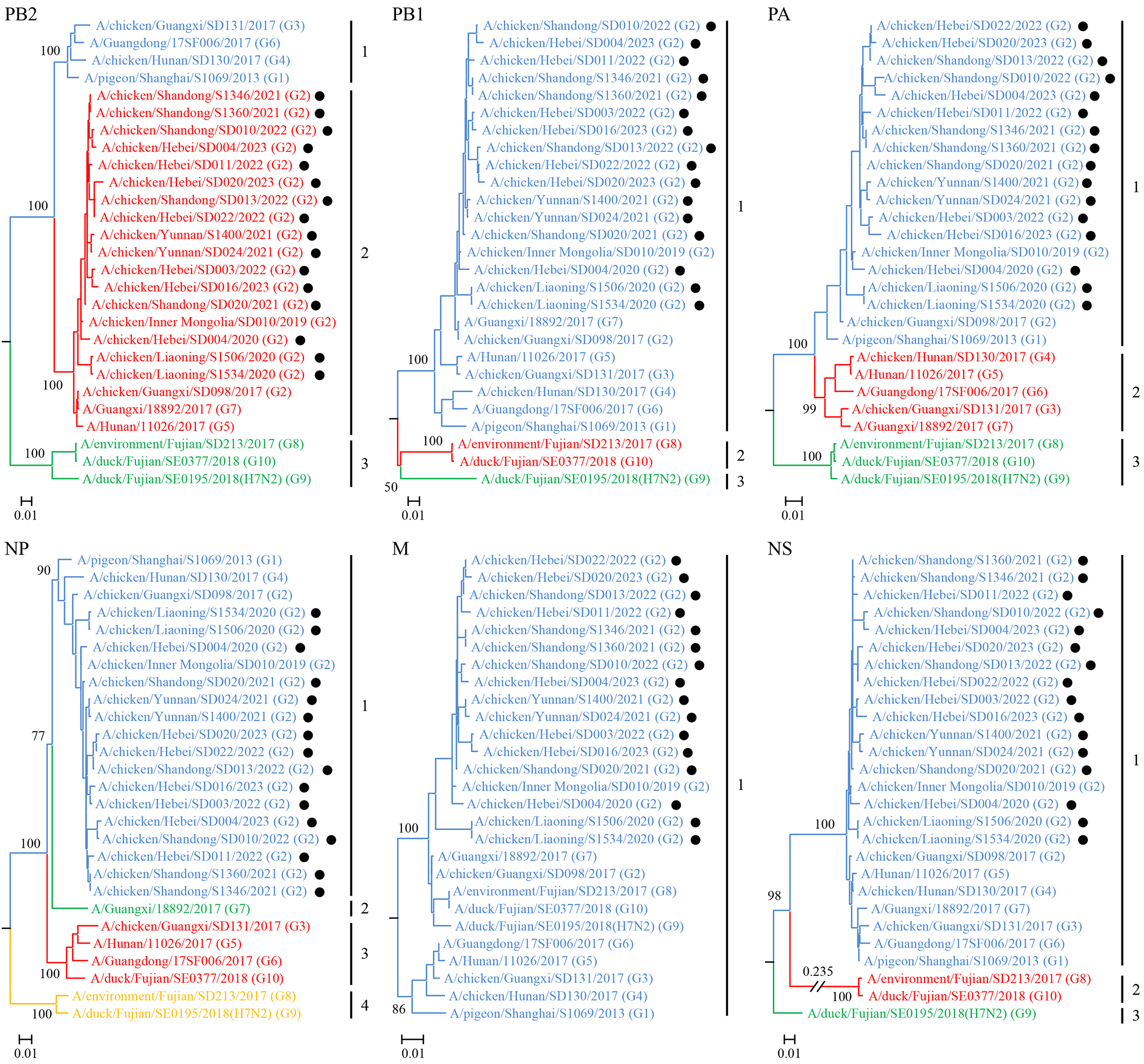

Supplement: updated_Supporting_figure_S1 [file TEMI_A_2343912_SM4027.tif]
