# Supplementary material for: Evolution of H7N9 highly pathogenic avian influenza virus in the context of vaccination
Source: Emerg Microbes Infect. 2024 Apr 17;13(1):2343912. doi: 10.1080/22221751.2024.2343912 (PMC11060016; doi:10.1080/22221751.2024.2343912)
Supplement: Hou_Table_S1 [file TEMI_A_2343912_SM5535.docx]

**Table S1.** Cross-reactive HI antibody titers of H7N9 viruses with the antisera induced by different vaccines*.

| **Virus (HA clade)** | **Antiserum induced by** | |
| --- | --- | --- |
|  | **H7-Re3** | **H7-Re4** |
| H7-Re3 | **512** | 256 |
| H7-Re4 | 64 | **1024** |
| CK/LN/S1506/2020 | 256 | 256 |
| CK/LN/S1534/2020 | 256 | 256 |
| CK/HeB/SD004/2020 | 64 | 128 |
| CK/SD/SD020/2021 | 128 | 512 |
| CK/SD/S1346/2021 | 32 | 256 |
| CK/SD/S1360/2021 | 32 | 256 |
| CK/YN/S1400/2021 | 32 | 512 |
| CK/YN/SD024/2021 | 32 | 512 |
| CK/HeB/SD003/2022 | 16 | 256 |
| CK/SD/SD010/2022 | 32 | 128 |
| CK/HeB/SD011/2022 | 32 | 256 |
| CK/SD/SD013/2022 | 16 | 64 |
| CK/HeB/SD022/2022 | 16 | 64 |
| CK/HeB/SD004/2023 | 32 | 64 |
| CK/HeB/SD016/2023 | 32 | 256 |
| CK/HeB/SD020/2023 | 16 | 64 |

*****, Homologous titers were shown in bold face and underlined.
